# Genetic Diversification of Starch Branching Enzymes during Maize Domestication and Improvement

**DOI:** 10.3390/genes14051068

**Published:** 2023-05-11

**Authors:** Qi Li, Tiantian Yang, Wenye Rui, Houmiao Wang, Yunyun Wang, Zefeng Yang, Chenwu Xu, Pengcheng Li

**Affiliations:** 1 Jiangsu Key Laboratory of Crop Genetics and Physiology/Key Laboratory of Plant Functional Genomics of the Ministry of Education/Jiangsu Key Laboratory of Crop Genomics and Molecular Breeding, Agricultural College of Yangzhou University, Yangzhou 225009, China; cookie11210914@163.com (Q.L.); yt2603360264@163.com (T.Y.); dx120210123@stu.yzu.edu.cn (W.R.); houmiaowang@yzu.edu.cn (H.W.); dx120180078@yzu.edu.cn (Y.W.); zfyang@yzu.edu.cn (Z.Y.); qtls@yzu.edu.cn (C.X.); 2 Jiangsu Co-Innovation Center for Modern Production Technology of Grain Crops, Yangzhou University, Yangzhou 225009, China; 3 Joint International Research Laboratory of Agriculture and Agri-Product Safety, Ministry of Education of China, Yangzhou University, Yangzhou 225009, China

**Keywords:** maize, starch branching enzyme, starch pasting and gelatinization properties, association analysis, linkage disequilibrium

## Abstract

Elucidating the genetic basis of starch pasting and gelatinization properties is crucial for enhancing the quality of maize and its utility as feed and industrial raw material. In maize, *ZmSBE* genes encode important starch branching enzymes in the starch biosynthesis pathway. In this study, we re-sequenced the genomic sequences of *ZmSBEI*, *ZmSBEIIa*, *ZmSBEIIb*, and *ZmSBEIII* in three lines called 335 inbred lines, 68 landrace lines, and 32 teosinte lines. Analyses of nucleotide polymorphisms and haplotype diversity revealed differences in the selection patterns of *ZmSBEI*, *ZmSBEIIa*, *ZmSBEIIb*, and *ZmSBEIII* during maize domestication and improvement. A marker–trait association analysis of inbred lines detected 22 significant loci, including 18 SNPs and 4 indels significantly associated with three maize starch physicochemical properties. The allele frequencies of two variants (SNP17249C and SNP5055G) were examined in three lines. The frequency of SNP17249C in *ZmSBEIIb* was highest in teosinte lines, followed by landrace lines, and inbred lines, whereas there were no significant differences in the frequency of SNP5055G in *ZmSBEIII* among the three lines. These results suggest that *ZmSBE* genes play an important role in the phenotypic variations in the starch physicochemical properties in maize. The genetic variants detected in this study may be used to develop functional markers for improving maize starch quality.

## 1. Introduction

Maize starch is one of the highest-quality starches and accounts for 80% of the global starch yield [1]. Moreover, it is used as food and feed as well as an industrial raw material (e.g., for biodiesel production) [2]. Therefore, identifying the genes or natural variations associated with starch quality and quantity in maize kernels may help to increase the nutritional value of maize through the breeding of high-quality lines.

Starch is an important storage polysaccharide comprising amylose and amylopectin, which are structurally diverse polymers. The content ratio of these two components influences the function, quality, and utility of starch. Changing the structure of starch may enhance how it can be used. The basic methods for synthesizing starch in plants include synthesizing soluble precursors, followed by coordinated reactions: firstly, the glucan chain is connected to α-(1→4) and elongating, and then, branching, and debranching at α-(1→6) positions (i.e., hydrolysis of specific branch linkages). AGPase synthesizes the nucleotide diphosphate sugar precursor ADP-Glc through the catalysis of ATP and Glc1P. The α-(1→4)-linked glucan chains are formed by reactions catalyzed by ADP-Glc-dependent transferases, which are called starch synthases (SSs), whereas α-(1→6)-linked branch points are introduced via reactions mediated by starch branching enzymes (SBEs) and debranching is catalyzed by debranching enzymes (DBEs) [3]. Thus, the starch synthesis pathway requires diverse enzymes (e.g., GBSSI, SSs, SBEs, and DBEs). Of these enzymes, GBSSI (granule-bound starch synthase I) synthesizes amylose in the cereal endosperm, while SSs, SBEs, and DBEs coordinately synthesize amylopectin. Two non-enzymatic proteins (PTST1 and PTST2) occur in the starch synthesis pathway. Notably, SBEs are the key enzymes catalyzing the synthesis of amylopectin, which is the main component of plant starch. Therefore, the nature and activity of SBEs are the decisive factors affecting the fine structure of amylopectin [4].

As the only enzymes that catalyze the formation of amylopectin branch linkages, which are called starch branching enzymes, they modify glucan to produce branches connected by α-1,6-glycosidic bonds [5]. On the basis of amino acid sequence relationships, SBEs have been divided into the following two classes: Class I (B family) and Class II (A family). Gene expression analyses of a range of species revealed that during development, *SBEI* genes are expressed later than *SBEII* genes [6]. In cereals, there are three types of SBE-encoding genes, namely *SBEI*, *SBEII*, and *SBEIII*. Earlier research indicated *SBEII* genes can be subdivided into *SBEIIa* and *SBEIIb* genes, which differ in terms of their expression kinetic characteristics and tissue expression patterns. Specifically, *SBEIIb* is expressed exclusively in the endosperm tissue, whereas *SBEIIa* appears to be expressed ubiquitously [7]. Moreover, *SBEII* is important for amylopectin synthesis in the cereal endosperm. However, there are considerable variations in the relative proportions of *SBEIIa* and *SBEIIb* activities in the endosperm among diverse cereals [7,8]. In the developing wheat endosperm, *SBEIIb* is expressed at much lower levels than *SBEIIa* [9], whereas in the maize endosperm, the *SBEIIb* expression level is approximately 50-times greater than *SBEIIa* [8]. Furthermore, *SBEIII*, which differs from *SBEI* and *SBEII*, has been detected in many higher plants, including rice, maize, and wheat, although only *TaSBEIII* contributes to the production of both A and B granules in wheat grains [10,11].

Maize was domesticated from teosinte approximately 9000 years ago. Kernel starch was one of the targets of artificial selection during maize domestication [12]. The starch biosynthesis-related functions of individual SBE isozymes have been examined, but *SBE* gene sequence polymorphisms and natural variations in maize remain unclear. It is also unknown whether *SBE* genes were selected during maize domestication and improvement. In this study, we re-sequenced four *ZmSBE* genes in 335 inbred lines, 68 landrace lines, and 32 teosinte lines. The objectives of this research were as follows: firstly, examine the diversity in the *ZmSBE* sequences among three lines; secondly, identify natural variations in candidate genes associated with the kernel starch content and starch pasting and gelatinization properties; finally, examine significant associations to clarify their involvement in maize domestication and improvement.

## 2. Materials and Methods

### 2.1. Plant Materials

A total of 335 inbred lines, 68 landrace lines, and 32 teosinte lines were selected in this study (Table S1) [13]. The inbred lines were planted in the experimental field at Sanya (N 18°23′, E 109°44′), Hainan, China, in 2017 and 2018. The plants were grown by the randomized complete block design (RCBD) with two replicates. Each inbred line was grown in a single row, with a length of 3 m and a distance between each row of 0.5 m.

### 2.2. DNA Isolation and ZmSBEs Resequencing

The genomic DNA of each line was extracted from three mixed leaves (approximately 15 days after the emergence of maize) using the CTAB (cetyl trimethyl ammonium bromide) method [14,15]. The sequences (B73_V3 reference genome) of the *ZmSBEI* (GRMZM2G088753), *ZmSBEIIa* (GRMZM2G073054), *ZmSBEIIb* (GRMZM2G032628), and *ZmSBEIII* (GRMZM2G005298) gene in all tested lines were sequenced using the target sequence capture sequencing technology on the NimbleGen platform [16,17] by BGI Life Tech Co., Ltd (Guangdong, China). The reference sequences captured by the target sequence were the *ZmSBEs* gene sequences in the background of the B73 inbred line (Table S2). 

### 2.3. Determination of Maize Kernel Starch Content and Starch Pasting and Gelatinization Properties

At the maturity stage, the ears for each inbred line were harvested, and approximately 50–60 grains were stripped from the middle of the corn ear and immediately frozen in liquid nitrogen, and preserved at −75 °C for analysis and determination of starch. After drying and weighing, the granules were crushed and passed through a sieve (100 mesh, d = 0.149 mm) to determine the starch content. The crude starch content of corn (KSC) kernel was estimated using a near-infrared analyzer (FOSS, Beijing, China).

A rapid visco analyser (RVA) (Model No. RVA-3D, Newport Scientific, Sydney, Australia) was used to determine the pasting properties. A total of 3 g of starch taken from each inbred line was dispersed into 25 mL of distilled water in the viscometer test canister. Then, the test was performed with RVA. The set speed was 160 rpm/min. Viscosity values were recorded in centipoise (cp). The pasting properties were peak viscosity (PV), trough viscosity (TV), breakdown viscosity (BD), final viscosity (FV), setback viscosity (SB), peak time (PT), and pasting temperature (P_temp_).

The gelatinization properties of maize starches were analyzed using a differential scanning calorimeter DSC 200F3 Maia (Netzsch, Germany). Firstly, 5 mg starch samples (dried starch basis) were weighed in a small crucible. Secondly, 10 mL of distilled water was added to mix it. Finally, it was sealed and stored at 4 °C. The samples were used the next day to perform DSC determination. The gelatinization properties were onset temperature (To), peak temperature (Tp), conclusion temperature (Tc), and the enthalpy of gelatinization (Δ*H_gel_*).

### 2.4. Analysis of Sequence Data

Multiple sequence alignments of the maize gene *ZmSBEs* were performed using MAFFT software [18]. MEGA7 software was used for manual proofreading. The analysis of sequences was performed using the software DNASP 5.0 [15,17]. The symbols π and θ denoted the nucleotide polymorphism of the gene, whereby π is the average number of nucleotide differences per site between any two DNA sequences, and θ was derived from the total number of segregating sites and corrected for sampling size. Tajima’s D [19] and Fu and Li’s D* and F* statistical tests [20] were used to test the neutral evolution. The levels of linkage disequilibrium (LD) between two polymorphic sites in a coding region were calculated using TASSEL 5.0 [21], and the LD level was expressed by the linkage disequilibrium parameter r^2^.

A total of 163,931 SNPs were obtained by genotyping using a sequencing strategy [13]. Among them, we removed the markers with a deletion rate of more than 20% and a minor allele frequency (MAF) of less than 1%. Principal component analysis (PCA) and kinship were calculated using TESSEL 5.0, and the top five PCs (Figure S1) were used to create a population structure matrix in 335 inbred lines. Gene-based variants with MAF ≥ 0.05 were identified by TASSEL 5.0. Association analysis was performed using mixed linear models (MLM) + principal components analysis (PCA) + Kinship in TASSEL 5.0. The *p* value threshold to control the genome-wide type 1 error rate was 2.1 × 10^−3^ (1/*n*, where *n* is 479, the largest marker number of 4 SBEs).

## 3. Results

### 3.1. ZmSBE Sequence Polymorphisms

**Figure 1:**
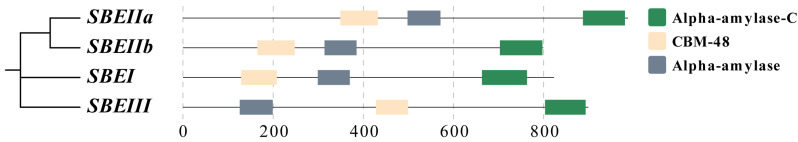
Protein domain of *ZmSBEs*. Each of the four gene domains contains α-amylase-C, CBM-48, and α-amylase, which are distinguished by different colors.

The analysis of four *SBE* isozyme-encoding genes revealed *ZmSBEI*, *ZmSBEIIa*, *ZmSBEIIb*, and *ZmSBEIII* differ substantially in terms of length (i.e., 5694, 10,473, 17,049, and 2940 bp, respectively) (Table 1). Both *ZmSBEI* and *ZmSBEIIb* were detected on chromosome 5, whereas *ZmSBEIIa* and *ZmSBEIII* were detected on chromosomes 2 and 8, respectively. The encoded amino acid sequences consisted of similar domains, with all four proteins containing α-amylase-C, CMB-48, and α-amylase (Figure 1). To identify the *ZmSBE* sequence polymorphisms, such as SNPs and indels, the upstream genomic region (approximately 2000 bp), coding region, and downstream genomic region (approximately 500 bp) in three tested lines were sequenced. For *ZmSBEI*, 1102 polymorphisms were found, consisting of 916 SNPs and 186 indels. On average, the SNPs and indels occurred every 9.9 and 50 bp, respectively. The average length of each indel was 5.4 bp. A total of 1519 sequence variations were identified in *ZmSBEIIa*, including 1302 SNPs and 217 indels. On average, the SNPs and indels were detected every 9.7 and 58.8 bp, respectively. The average length of each indel was 4.9 bp. Of the 1616 sequence variations detected in *ZmSBEIIb*, 1353 were SNPs and 263 were indels. On average, the SNPs and indels occurred every 14.5 and 76.9 bp, respectively. The average length of each indel was 3.9 bp. In contrast, *ZmSBEIII* included 1044 polymorphisms, including 903 SNPs and 141 indels. On average, the SNPs and indels were detected every 7.6 and 47.6 bp, respectively. The average length of each indel was 4.7 bp (Table 1).

### 3.2. Analysis of Nucleotide Diversity and Selection of ZmSBE Genes in Teosinte, Landraces, and Inbred Lines

**Figure 2:**
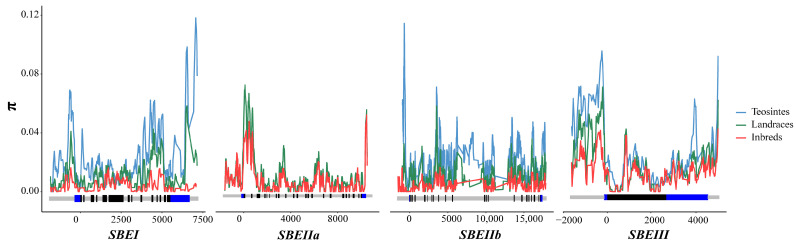
The nucleotide diversity (π) of teosinte, landraces, and inbred lines. π is calculated using the method of sliding windows of 100 bp with a step of 25 bp. A schematic diagram of the *ZmSBEs* gene structure, which contains upstream sequence and introns (light gray), the coding region (black), and 5’ UTR and 3’ UTR (blue) are shown. The position of the start codon (ATG) is labeled as “0”, and the negative value indicates the upstream sequence of the gene.

To survey the variety among the four *ZmSBE* genes (*ZmSBEI*, *ZmSBEIIa*, *ZmSBEIIb*, and *ZmSBEIII*) in all tested lines, sequence variations were analyzed. Compared with the genes in the teosinte lines, those in the landrace lines and inbred lines were more conserved (C_T_ < C_L_ < C_I_), less diverse (π_T_ > π_L_ > π_I_), and had fewer nucleotide sequence polymorphisms (θ_T_ > θ_L_ > θ_I_) (Table 2). The π value was lower for *ZmSBEIIa* than for the other three genes, implying that *ZmSBEIIa* had the least diverse nucleotide sequence (Table 2 and Figure 2). To further assess whether *ZmSBE* genes were chosen during maize evolution, neutrality tests (Tajima’s D along with Fu and Li’s D* and F*) were performed to analyze the tested sequences. Tajima’s D and Fu and Li’s F* values for *ZmSBEI* and *ZmSBEIIb* were significantly less than 0 in all test lines, showing that these two genes were chosen during evolution. For both *ZmSBEIIa* and *ZmSBEIII*, Fu and Li’s D* and F* values were significantly less than 0, but Tajima’s D value was not. Hence, these two genes may have undergone neutral evolution (Table 2).

**Figure 3:**
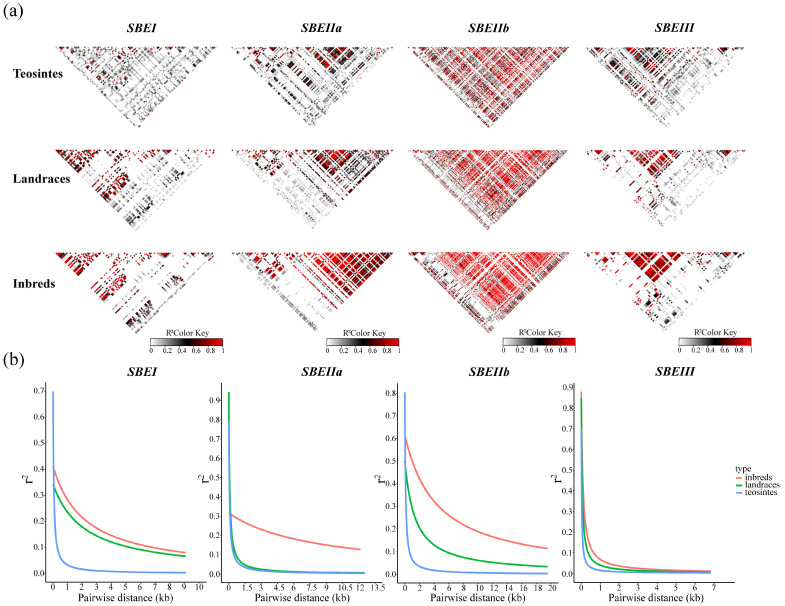
Linkage disequilibrium and recombination events. (**a**) Linkage disequilibrium analysis of *ZmSBEs* for three lines. It indicates LD between pairs of *ZmSBEs* sequence polymorphic sites. The R^2^ values are indicated using the color bar. (**b**) LD decay of *ZmSBEs* for three lines determined by r^2^. Three lines are inbred lines (red), landrace lines (green), and teosinte lines (blue).

Linkage disequilibrium (LD) decay varied among the analyzed genes. For *ZmSBEI*, LD was less extensive in the teosinte lines than in the landrace lines and inbred lines, implying that this gene may have been chosen during maize domestication. For *ZmSBEIIa*, the extent of LD in the teosinte lines was similar to that in the landrace lines, but lower than in the inbred lines. Accordingly, *ZmSBEIIa* may have been chosen during maize improvement. In contrast, *ZmSBEIIb* may have been chosen during both maize domestication and improvement, whereas the likelihood that *ZmSBEIII* was chosen during maize domestication and improvement was relatively low (Figure 3).

### 3.3. Analysis of the Association between Phenotypes and ZmSBEs

**Figure 4:**
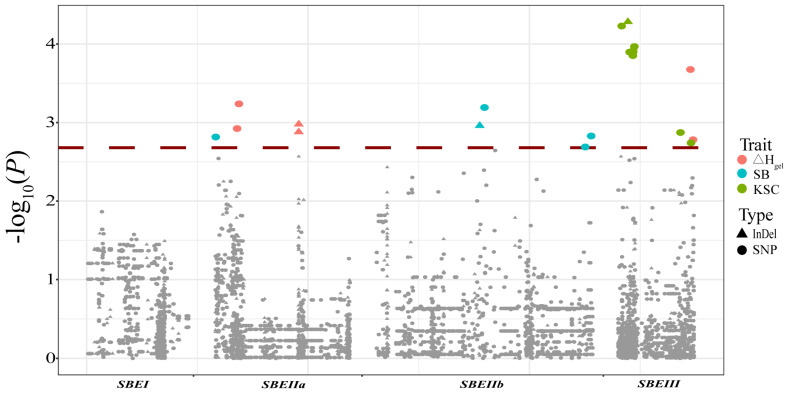
Association analysis between *ZmSBEs* and starch quality-related traits. Indels are marked by triangles and SNPs are signed by dots. Trait abbreviations: Δ*H_gel_*: the enthalpy of gelatinization; SB: setback viscosity; KSC: crude starch content.

A total of 12 maize starch physicochemical properties were analyzed in 335 inbred lines (Table S3). The phenotypic fold of variation and coefficient of variation had a wide range of parameters. These results reflected the considerable differences in the phenotypes of the inbred lines (Table S1). To investigate whether the natural variations in *ZmSBE* sequences were associated with starch physicochemical properties, an association analysis was performed on the basis of the variants with a minor allele frequency ≥ 0.05. Twenty-two significant variants of *ZmSBEIIa*, *ZmSBEIIb*, and *ZmSBEIII* were associated with three traits (Figure 4 and Table 3). Five significant loci in *ZmSBEIIa* were associated with Δ*H_gel_* and SB, and the phenotypic variation explained ranged from 2.99% to 4.28%. The variation in Δ*H_gel_* was mainly explained by indel 15969 in the intron region. Four significant loci in *ZmSBEIIb* were associated with SB, and the phenotypic variation explained ranged from 2.83% to 3.95%. Thirteen significant loci associated with Δ*H_gel_* and KSC were detected in *ZmSBEIII*, and the phenotypic variation explained ranged from 2.82% to 4.88%. The phenotypic variation was mainly explained by indel 830 in the upstream region. We also observed that individual traits were significantly associated with different genes. For example, Δ*H_gel_* and SB were controlled by *ZmSBEIIa* and *ZmSBEIII* (Table 3).

**Figure 5:**
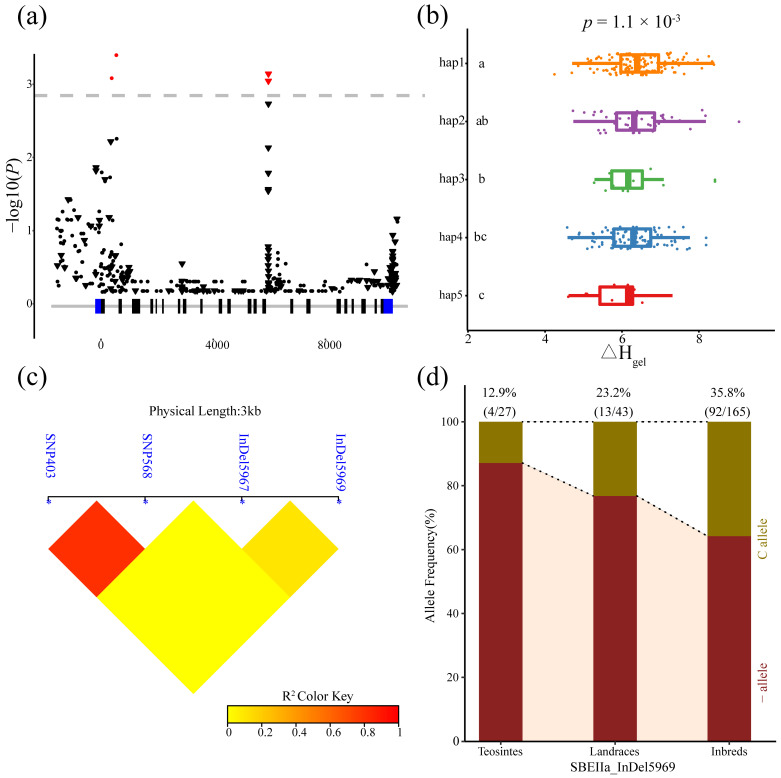
Natural variability in *ZmSBEIIa* was significantly correlated with enthalpy (Δ*H_gel_*). (**a**) The Manhattan Plot of the association of *ZmSBEIIa* and Δ*H_gel_*. A diagrammatic sketch of the *ZmSBEIIa* gene structure is shown. (**b**) Comparisons of Δ*H_gel_* between haplotypes carrying different alleles in inbred lines. (**c**) Linkage disequilibrium (LD) analysis of four significant variants associated with Δ*H_gel_*. (**d**) The allele frequency of indel 5969 in three tested lines.

The four loci in *ZmSBEIIa* significantly associated with Δ*H_gel_* (403, 568, 5967, and 5969 bp) consisted of two SNPs and two indels. These loci were in intronic regions. The analysis of these variants detected strong LD for SNP403 and SNP568. The inbred lines were divided into five haplotypes according to the significant variants. The ANOVA results indicated that the differences in Δ*H_gel_* among the five haplotypes were significant (*p* = 1.1 × 10^−3^). We also analyzed the frequency of indel 5969 (i.e., the most significant variant) in teosinte, landraces, and inbred lines. The results indicated that the frequency of indel 5969C was lower in the teosinte lines (12.9%) than in the landrace lines (23.2%), and inbred lines (35.8%). Accordingly, indel 5969 may have been chosen during maize domestication and improvement (Figure 5).

**Figure 6:**
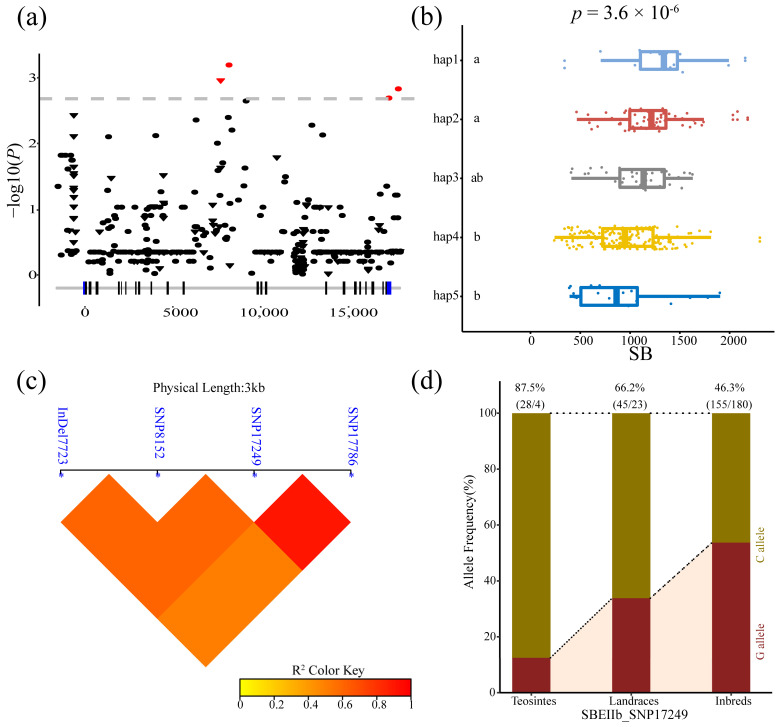
Natural variations in *ZmSBEIIb* were significantly correlated with setback viscosity (SB). (**a**) The Manhattan Plot of the association of *ZmSBEIIb* and SB. A diagrammatic sketch of the *ZmSBEIIb* gene structure is shown. (**b**) Comparisons of SB between haplotypes carrying different alleles in inbred lines. (**c**) Linkage disequilibrium (LD) analysis of four significant variants associated with SB. (**d**) The allele frequency of SNP 17249 in three tested lines.

Four variants of *ZmSBEIIb* were associated with SB, including one indel and three SNPs (7723, 8152, 17,249, and 17,786 bp). The LD analysis showed that SNP17249 and SNP17786 are completely linked; the LD value (r^2^) of the remaining loci was approximately 0.8. Five major haplotypes were identified for the four loci across the 335 inbred lines. The differences in SB among these haplotypes were significant according to ANOVA (*p* = 1.1 × 10^−6^). The frequency of SNP 17249C was higher in the teosinte lines (87.5%) than in the landrace lines (66.2%), and inbred lines (46.3%), which indicated that SNP 17249C may have been gradually selected during maize domestication and improvement (Figure 6).

**Figure 7:**
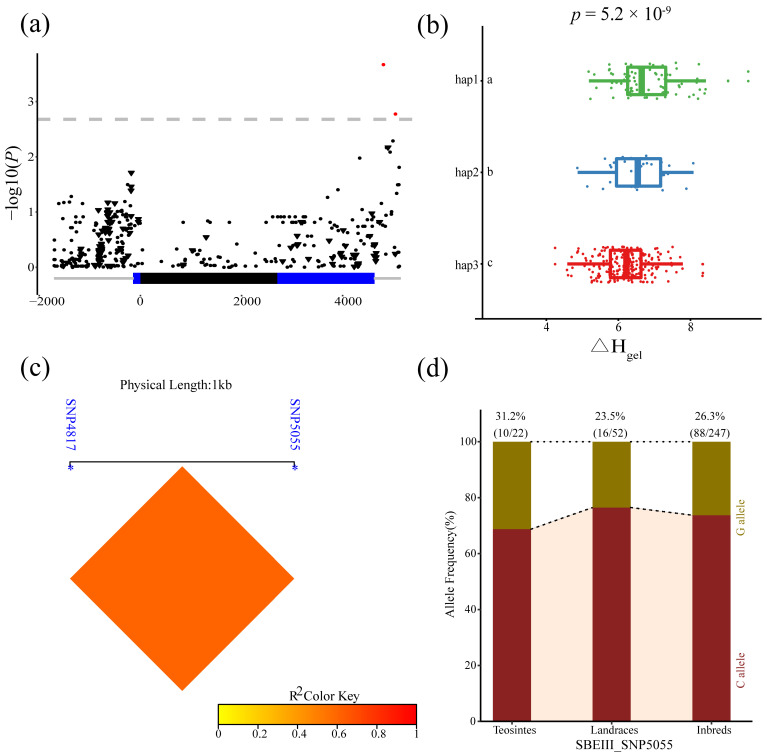
Natural variations in *ZmSBEIII* were significantly correlated with enthalpy (Δ*H_gel_*). (**a**) The Manhattan Plot of the association of *ZmSBEIII* and Δ*H_gel_*. A diagrammatic sketch of the *ZmSBEIII* gene structure is shown. (**b**) Comparisons of SB between haplotypes carrying different alleles in inbred lines. (**c**) Linkage disequilibrium (LD) analysis of four significant variants associated with Δ*H_gel_*. (**d**) The allele frequency of SNP 5055 in three tested lines.

Two SNPs in *ZmSBEIII* (4817 and 5055 bp) were significantly associated with Δ*H_gel_*. Strong LD was observed between these two loci. In addition, significant differences in Δ*H_gel_* were detected among the three haplotypes for these two loci (*p* = 5.2 × 10^−9^). The frequency of SNP5055G did not differ significantly among the three populations, indicating that SNP5055 is not exposed to obvious selection during maize domestication and improvement (Figure 7).

## 4. Discussion

Significant phenotypic differences and nucleotide polymorphisms are important for genetic mapping through linkage or association analyses [22,23]. Maize is a typical outcrossing crop, which has broad morphological variations, genetic diversity, and a high effective recombination rate [24,25]. The plant breeders can create novel plant gene combinations via abundant genetic variations and choose the variety of crops appropriate for diverse agricultural systems [22,23]. Analyzing the genetic diversity to screen for functional genes is critical for clarifying the genetic basis of crop phenotypic variations, thereby generating relevant information for crop improvement [23,26]. In this study, we revealed the polymorphisms in maize *ZmSBE* genes, which encode important enzymes in the starch biosynthesis pathway, in 335 inbred lines, 68 landrace lines, and 32 teosinte lines. The average nucleotide diversities (π) of these four genes were higher than previously reported in the whole genome [27]. The inbred lines had 52.0–82.8% less diversity than teosintes, and the selected gene *ZmMADS69*, which functions as a flowering activator, only retained 18.4% of the nucleotide diversity of teosinte [28]. In rice and soybean, cultivated varieties retained 70% and 36% of the nucleotide diversity of wild varieties [29,30]. Among these genes, *ZmSBEIIb* had the most variant sites and the highest variant frequency. Candidate gene association mapping on the basis of LD is a powerful method for identifying elite alleles for target traits [25]. This approach has been used to analyze several genes to detect variants associated with kernel composition [22,31,32,33]. In a previous study, markers were developed for the most favorable alleles of *crtRB1*, which is associated with the maize kernel β carotene concentration, for the provitamin A biofortification of crops [32]. In the current study, 22 significant variants of *ZmSBEIIa*, *ZmSBEIIb*, and *ZmSBEIII* were related to KSC, Δ*H_gel_*, and SB. The elite variations and the best haplotypes of *ZmSBEIIa*, *ZmSBEIIb*, and *ZmSBEIII* were identified, which may be used to generate inexpensive markers useful for enhancing the starch quality and quantity by molecular breeding in maize.

Compared with teosinte, cultivated maize has undergone considerable phenotypic changes (e.g., plant, ear, and seed morphologies) [34]. It is estimated that 2–4% of the genes were chosen during maize domestication and improvement [35]. There are also differences in kernel composition between teosinte and modern maize varieties. Starch is the main component of cereal seeds and contributes substantially to grain yield. The kernel starch content has been altered via strong selection during domestication and plant breeding [12]. The maize kernel starch content of inbred lines is reportedly 71.7%, which is higher than the corresponding content in teosinte kernels (52.92%) [12]. Previous research revealed that starch metabolism is the primary pathway in maize and rice that underwent convergent selection [36]. A total of 11 orthologous gene pairs in the starch metabolic pathway are associated with convergent selection. The *ZmSBEI* gene was chosen in both rice and maize [36]. The four *ZmSBE* genes in our study have different selection patterns during maize domestication and improvement. Specifically, *ZmSBEI* may have been chosen during maize domestication, while *ZmSBEIIa* may have been chosen during maize improvement. In contrast, it is possible that *ZmSBEIIb* was chosen during both maize domestication and improvement, whereas there was likely minimal selection of *ZmSBEIII* during maize domestication and improvement. Differences in the evolution of *z2* genes, which influence the maize seed *zein* content, have been reported. The results revealed that teosinte and landrace lines may be crucial sources of genetic variation relevant for maize improvement [37].

In conclusion, maize *ZmSBE* genes were re-sequenced in 335 inbred lines, 68 landrace lines, and 32 teosinte lines. Analyses of nucleotide polymorphisms and haplotype diversity revealed differences in the selection patterns of *ZmSBEI*, *ZmSBEIIa*, *ZmSBEIIb*, and *ZmSBEIII* during maize domestication and improvement. A total of 22 significant variants of *ZmSBEIIa*, *ZmSBEIIb*, and *ZmSBEIII* were identified as associated with KSC, Δ*H_gel_*, and SB. The effect and selection of elite variations of *ZmSBEIIa*, *ZmSBEIIb*, and *ZmSBEIII* were identified. These results suggest that the genetic diversity of *ZmSBE* genes plays an important role in the variations of the starch physicochemical properties in maize. These variants may be applicable for increasing the maize kernel starch quality and quantity.

## Figures and Tables

**Table 1:** Overview of parameters of the nucleotide polymorphisms analysis of *ZmSBEs*.

Parameters	*SBEI.*	*SBEIIa*	*SBEIIb*	*SBEIII*
Chr	5	2	5	8
Gene length	5694	10,473	17,049	2940
Total length of amplicons (bp)	9105	12,656	19,561	6831
Number of all of the sequence variants	1102	1519	1616	1044
Frequency of all of the sequence variants	8.3	8.3	12.0	6.5
Number of nucleotide substitutions (bp)	916	1302	1353	903
Frequency of polymorphic sites per bp	9.9	9.7	14.5	7.6
Number of indels	186	217	263	141
Frequency of indels per bp	50.0	58.8	76.9	47.6
Average indels length	5.4	4.9	3.9	4.7

**Table 2:** Summary of nucleotide polymorphisms and neutrality test of four *SBE* genes.

Gene	Pop.	C	*π*	*θ*	Tajima’s D	D*	F*
*SBEI*	Inbreds	0.952	0.00397	0.00583	−0.981	−0.739	−1.023
	Landraces	0.943	0.01098	0.01075	0.074	−0.324	−0.194
	Teosintes	0.870	0.02304	0.03078	−0.974	−1.271	−1.387
	ALL	0.843	0.00711	0.02005	−1.976 *	−6.655 **	−4.700 **
*SBEIIa*	Inbreds	0.948	0.00737	0.00734	0.010	−1.569	−0.850
	Landraces	0.943	0.01031	0.0108	−0.161	−0.988	−0.780
	Teosintes	0.879	0.01874	0.02777	−1.260	−1.614	−1.772
	ALL	0.862	0.00853	0.01892	−1.685	−7.167 **	−4.747 **
*SBEIIb*	Inbreds	0.948	0.00443	0.00533	−0.519	−2.571 *	−1.738
	Landraces	0.931	0.01085	0.01179	−0.283	−1.297	−1.058
	Teosintes	0.882	0.02117	0.0269	−0.826	−1.360	−1.397
	ALL	0.847	0.00725	0.01749	−1.797 *	−5.744 **	−4.095 **
*SBEIII*	Inbreds	0.915	0.01325	0.01143	0.494	0.271	0.459
	Landraces	0.889	0.01906	0.02012	−0.186	−0.828	−0.679
	Teosintes	0.826	0.02758	0.04001	−1.203	−1.222	−1.443
	ALL	0.792	0.01467	0.02709	−1.404	−5.439 **	−3.702 **

* and ** indicate the statistical significance separately at *p* < 0.05 or 0.01 level. Trait abbreviations: Pop, population; ALL, teosinte, landraces, and inbred lines; C, sequence conservation; D*, Fu & Li’s D*; F*, Fu & Li’s F*.

**Table 3:** Results of candidate gene-based association analysis of SBE genes.

Gene	Trait	Section	Variant	Genotype	*p*-Value	Contribution
*SBEIIa*	Δ*H_gel_*	Intron 14	Indel 5967	-/C	1.32 × 10^−3^	3.17%
*SBEIIa*	Δ*H_gel_*	Intron 14	Indel 5969	-/C	1.05 × 10^−3^	4.28%
*SBEIIa*	Δ*H_gel_*	Intron 1	SNP 403	G/T	1.19 × 10^−3^	3.21%
*SBEIIa*	Δ*H_gel_*	Intron 1	SNP 568	G/A	5.78 × 10^−4^	3.62%
*SBEIIa*	SB	Upstream	SNP 1529	T/C	1.53 × 10^−3^	2.99%
*SBEIIb*	SB	Intron 11	Indel 7723	-/C	1.10 × 10^−3^	3.39%
*SBEIIb*	SB	3’ UTR	SNP 17249	C/G	2.05 × 10^−3^	2.83%
*SBEIIb*	SB	Downstream	SNP 17786	T/A	1.48 × 10^−3^	3.01%
*SBEIIb*	SB	Intron 11	SNP 8152	A/G	6.42 × 10^−4^	3.95%
*SBEIII*	Δ*H_gel_*	Downstream	SNP 4817	T/C	2.11 × 10^−4^	4.21%
*SBEIII*	Δ*H_gel_*	Downstream	SNP 5055	C/G	1.65 × 10^−3^	3.02%
*SBEIII*	KSC	Upstream	Indel 830	G/-	5.20 × 10^−5^	4.88%
*SBEIII*	KSC	Upstream	SNP 1391	G/A	5.89 × 10^−5^	4.80%
*SBEIII*	KSC	Upstream	SNP 245	G/A	1.08 × 10^−4^	4.39%
*SBEIII*	KSC	Upstream	SNP 356	C/T	1.27 × 10^−4^	4.30%
*SBEIII*	KSC	Upstream	SNP 357	C/A	1.22 × 10^−4^	4.32%
*SBEIII*	KSC	Upstream	SNP 381	C/T	1.40 × 10^−4^	4.26%
*SBEIII*	KSC	Upstream	SNP 443	C/T	1.27 × 10^−4^	4.30%
*SBEIII*	KSC	Upstream	SNP 674	C/T	1.27 × 10^−4^	4.30%
*SBEIII*	KSC	3’ UTR	SNP 3918	A/T	1.34 × 10^−3^	2.99%
*SBEIII*	KSC	Downstream	SNP 4867	C/T	1.82 × 10^−3^	2.82%
*SBEIII*	KSC	Downstream	SNP 4868	C/T	1.82 × 10^−3^	2.82%

Trait abbreviations: Δ*H_gel_*: the enthalpy of gelatinization; SB: setback viscosity; KSC: crude starch content.

## Data Availability

The data sets supporting the results of this article are included within the article (and its Supplementary Files).

## Supplementary Materials

The following supporting information can be downloaded at: https://www.mdpi.com/article/10.3390/genes14051068/s1, Table S1: The list of 335 inbred lines, 68 landraces, and 32 teosinte lines used in this study; Table S2: The sequences of *ZmSBEs*; Table S3: Statistical analysis of 12 phenotypic traits for tested inbred lines; Figure S1: The effect of each principal component; Figure S2: Natural variability in *ZmSBEIIa* were significantly correlated with setback viscosity; Figure S3: Natural variability in *ZmSBEIII* were significantly correlated with starch.
