# Supplementary material for: Genetic Diversification of Starch Branching Enzymes during Maize Domestication and Improvement
Source: Genes (Basel). 2023 May 11;14(5):1068. doi: 10.3390/genes14051068 (PMC10218289; doi:10.3390/genes14051068)
Supplement: Supplementary file 1 [file genes-14-01068-s001.zip › Supplementary Table and Figure.pdf]

Supplementary Materials

Journal Name: Genes

Genetic Diversification of Starch Branching Enzymes during Maize Domestication and Improvement

Qi Li[1], Tiantian Yang[1], Wenye Rui[1], Houmiao Wang[1,2], Yunyun Wang[1], Zefeng Yang[1,2,3], Chenwu Xu[1,2,3], Pengcheng Li[1,2*]

Jiangsu Key Laboratory of Crop Genetics and Physiology/ Key Laboratory of Plant Functional Genomics of the Ministry of Education/ Jiangsu Key Laboratory of Crop Genomics and Molecular Breeding, Agricultural College of Yangzhou University, Yangzhou 225009, China;

* Corresponding authors: Pengcheng Li

Email: pcli@yzu.edu.cn

Tel.: +86-0514-87979358

Fax: +86-0514-87996817

Supplementary Table

Table S1 The list of 335 inbred lines, 68 landraces and 32 teosinte lines used in this study

Table S2 The sequences of *ZmSBEs*

Table S3 Statistical analysis of 12 phenotypic traits for tested inbred lines

Supplementary Figure

Figure S1 The effect of each principal component.

Figure S2 Natural variability in *ZmSBEIIa* were significantly correlation with setback viscosity

Figure S3 Natural variability in *ZmSBEIII* were significantly correlation with starch

Table S1 The list of 335 inbred lines, 68 landraces and 32 teosinte lines used in this study

Excel **Table S1**

Table S2 The sequences of *ZmSBEs*

Excel **Table S2**

Table S3 Statistical analysis of 12 phenotypic traits for tested inbred lines

Trait	Max	Min	Range	Mean	STD	CV	Fold
PV	3180	364	2816	1162.3	387.8	33.40%	8.74
TV	1494	339	1454	954.6	228.8	24.00%	4.41
BD	2006	5	2001	207.8	203.7	98.10%	401.2
FV	3509	623.5	3291.5	2007.7	492.7	24.50%	5.63
SB	2104	284.5	2405	1053.2	384.6	36.50%	7.4
PT	7	4.7	2.6	5.5	0.5	8.20%	1.49
Ptemp	93.9	78.7	17.6	82.5	2.8	3.40%	1.19
$\triangle H_{gel}$	8.5	3.2	6.4	6.4	0.8	13.00%	2.61
To	73.1	66.3	7.3	69.4	1.2	1.80%	1.1
Tp	78.9	72.9	6.7	75.1	1.1	1.40%	1.08
Tc	87.1	80	8.6	82.3	1.2	1.50%	1.09
KSC	69.4	61.6	10	67.4	1.3	1.90%	1.13

Trait abbreviations: PV, peak viscosity; TV, trough viscosity; BD, breakdown viscosity; FV, final viscosity; SB, setback viscosity; PT, peak time; Ptemp, pasting temperature; △Hgel, the enthalpy of gelatinization; To, onset temperature; Tp, peak temperature; Tc, conclusion temperature; KSC, the crude starch content of corn.

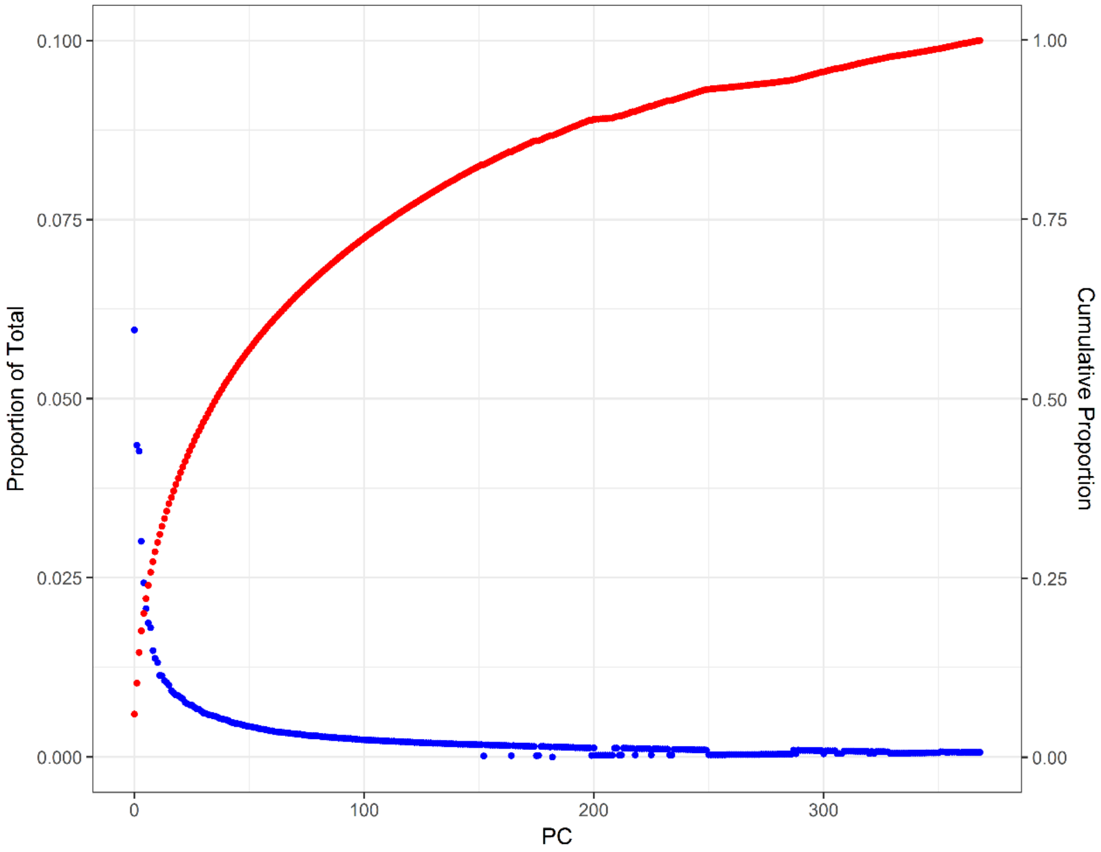

Figure S1 Plot of the effect of each principal component.

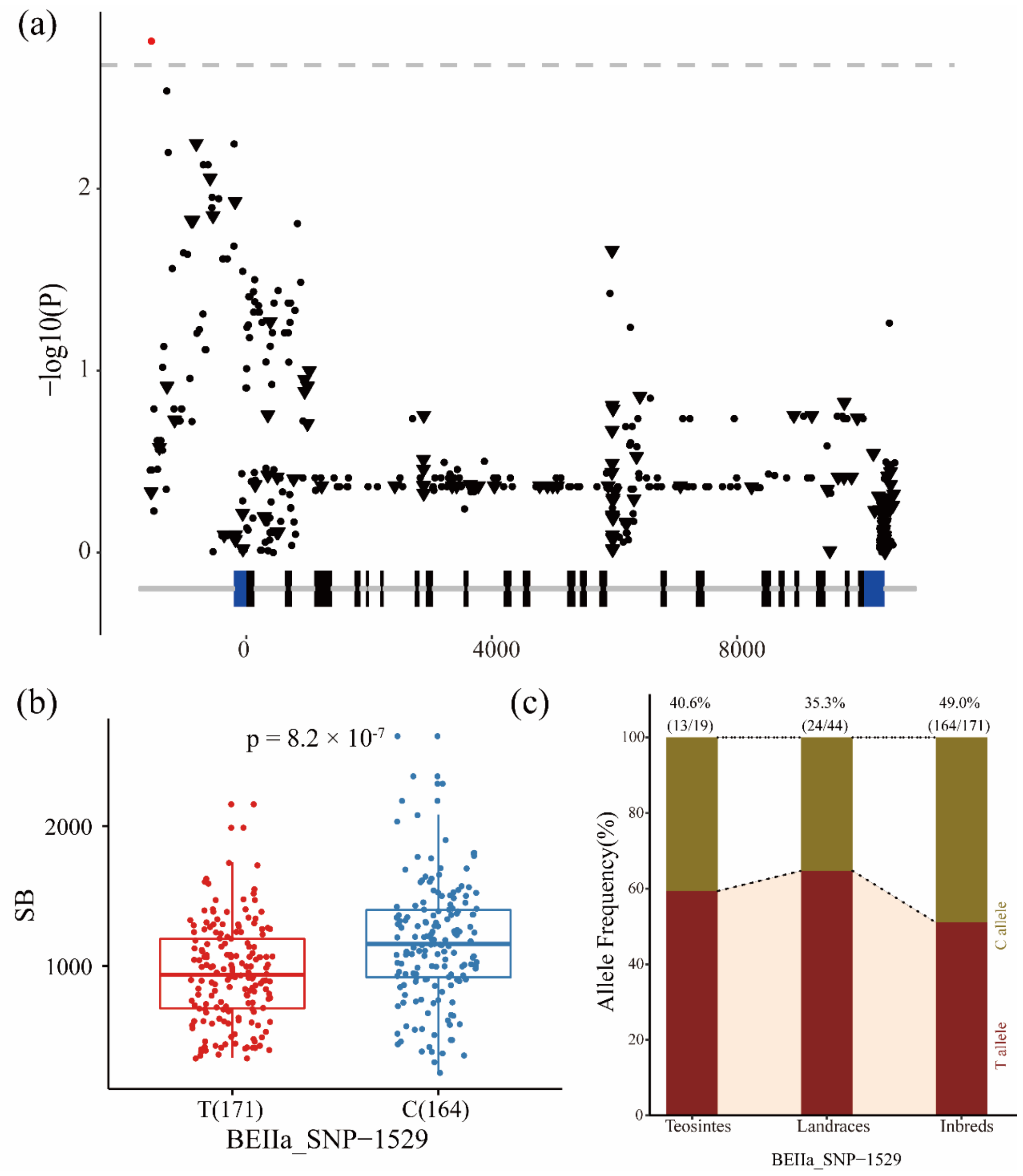

Figure S2 Natural variability in *ZmSBEIIa* were significantly correlation with setback viscosity (SB). (a) The Manhattan Plot of the association of *ZmSBEIIa* and SB. A diagrammatic sketch of the *ZmSBEIIa* gene structure is shown. (b) Comparisons of SB between different types of variation in inbred lines. (c) The allele frequency of SNP-1529 in three tested lines.

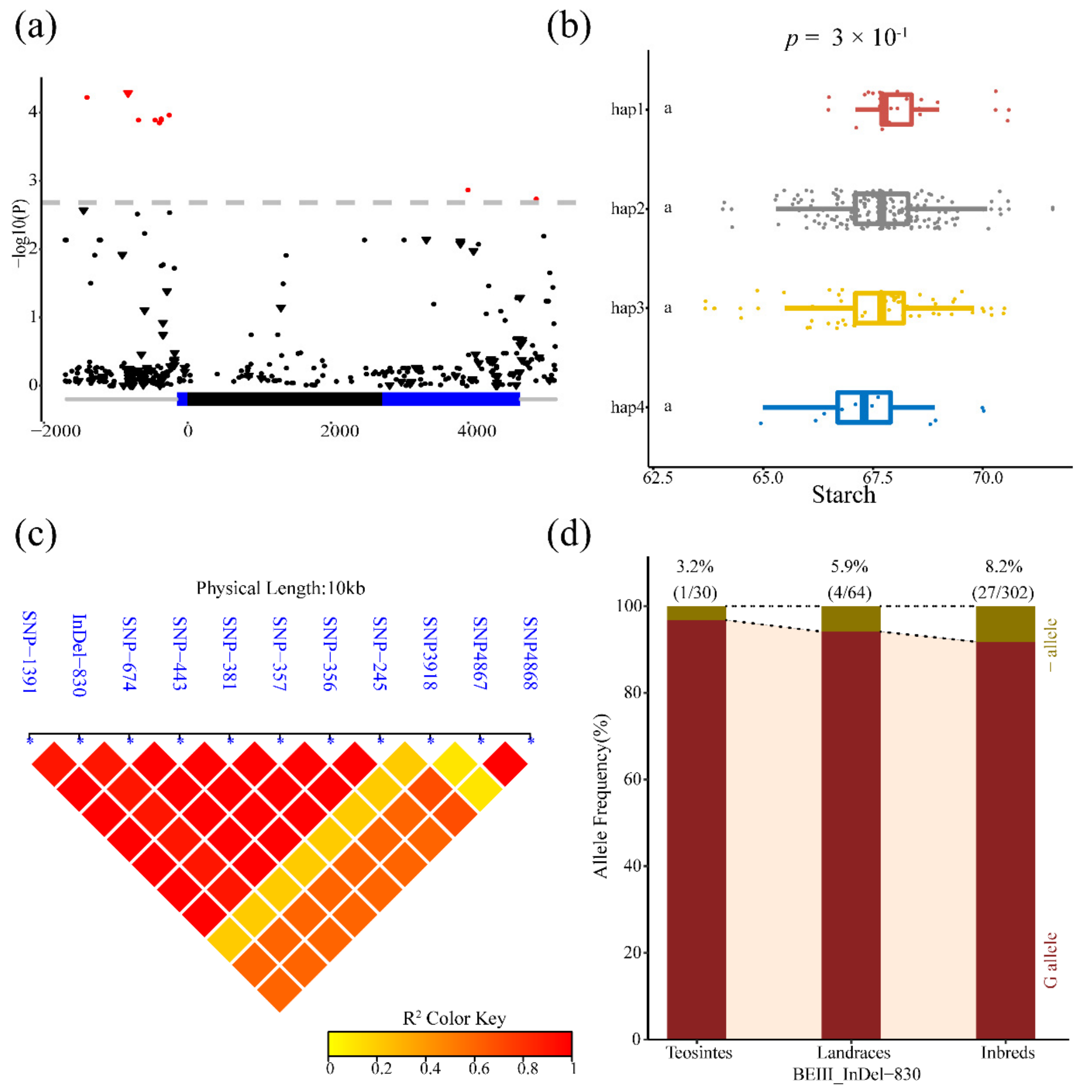

Figure S3 Natural variability in *ZmSBEIII* were significantly correlation with starch. (a) The Manhattan Plot of the association of *ZmSBEIII* and starch. A diagrammatic sketch of the *ZmSBEIII* gene structure is shown. (b) Comparisons of starch between haplotypes carrying different alleles in inbred lines. (c) Linkage disequilibrium (LD) analysis of eleven significant variants associated with starch. (d) The allele frequency of InDel-830 in three tested lines.
